# Supplementary material for: Unreprogrammed H3K9me3 prevents minor zygotic genome activation and lineage commitment in SCNT embryos
Source: Nat Commun. 2023 Aug 9;14:4807. doi: 10.1038/s41467-023-40496-3 (PMC10412629; doi:10.1038/s41467-023-40496-3)
Supplement: Supplementary file 1 — Supplementary Information [file 41467_2023_40496_MOESM1_ESM.pdf]

Supplementary Figures

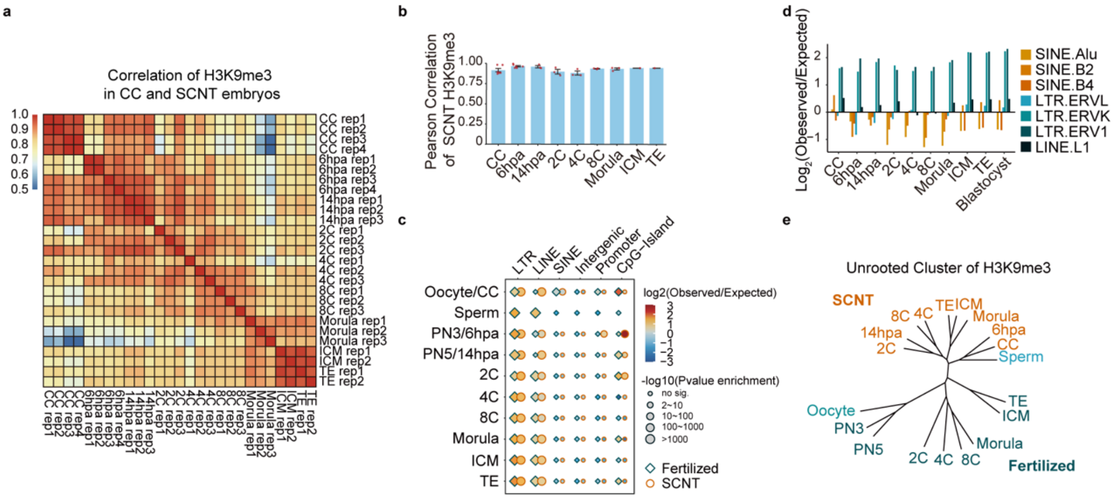

Supplementary Figure 1 Validation of data quality and genome-wide distribution of H3K9me3 in SCNT embryos. (a) Pearson correlation between samples in this study. **(b)** The Pearson correlations of ChIP-seq RPKM between replicates of H3K9me3. The number of biologically independent samples: n=4 for CC and 6hpa; n=3 for 14hpa, 2C, 4C, 8C and morula; n=2 for ICM and TE. **(c)** The enrichment of H3K9me3 peaks in LTR, LINE, SINE, intergenic, promoter and CpG-Island regions. **(d)** The enrichment of H3K9me3 peaks in Alu, B2 and B4 of SINE, ERVL, ERVK and ERV1 of LTR, LINE1 of LINE. **(e)** Unrooted dendrogram of H3K9me3 signal between fertilized and SCNT embryos derived from Hierarchical cluster analysis. **(b).** Data are presented as mean values ± SEM. Source data are provided as a Source Data file.

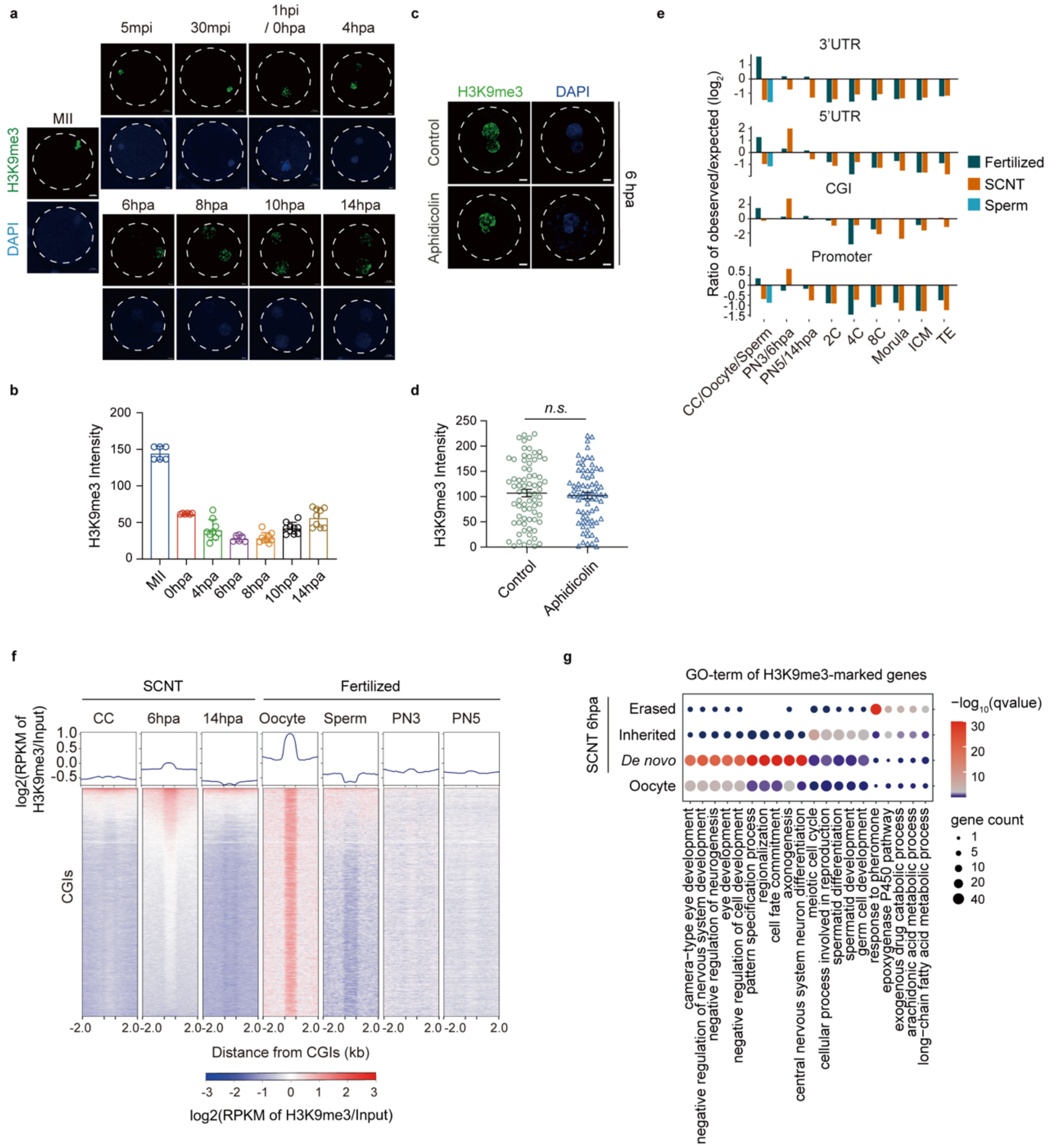

Supplementary Figure 2. H3K9me3 is globally lost at 6hpa and *de novo* H3K9me3 is enriched in CGI, similar to oocytes. (a) Immunostaining of H3K9me3 (green) in MII oocytes and SCNT embryos at 5mpi, 30mpi, 1hpi/0hpa, 4hpa, 6hpa, 8hpa, 10hpa and 14hpa. Each dotted lines circle a whole embryo. DAPI stains for DNA (blue). Scale bar, 10μm. mpi, minutes post injection. hpa, hour (s) post activation. **(b)** Quantification of fluorescence intensity of H3K9me3 signal in SCNT embryos in **a**. n = 6, 6, 9, 6, 9, 9, 9 for respective time points. **(c)** Immunostaining of H3K9me3 (green) in 6 hpa SCNT embryos with treatment of aphidicolin from 0hpa. Untreated embryos were used as control. DAPI stains for DNA (blue). Scale bar, 10μm. **(d)** Quantification of fluorescence intensity of H3K9me3 signal in 6hpa SCNT embryos in C. n = 72, 73 for control and aphidicolin treatment groups. **(e)** The enrichment of H3K9me3 peaks in 3'UTR, 5'UTR, CpG-islands and promoters. **(f)** The distribution of H3K9me3 signal around CpG-islands (CGIs) of CC, 6hpa, 14hpa, oocyte, sperm, PN3 and PN5 embryos. **(g)** The gene ontology (GO) analysis of genes marked by H3K9m3 at oocyte, and genes marked by *de novo*, inherited and erased H3K9me3 at 6hpa. The color of circles represent -log10 transformed q-value, and the size indicate the number of genes included. **(b, d).** Data are presented as mean values ± SEM. **(d).** Statistical significance was

calculated with a two-tailed Student's t test. ns, no significance. Source data are provided as a Source Data file.

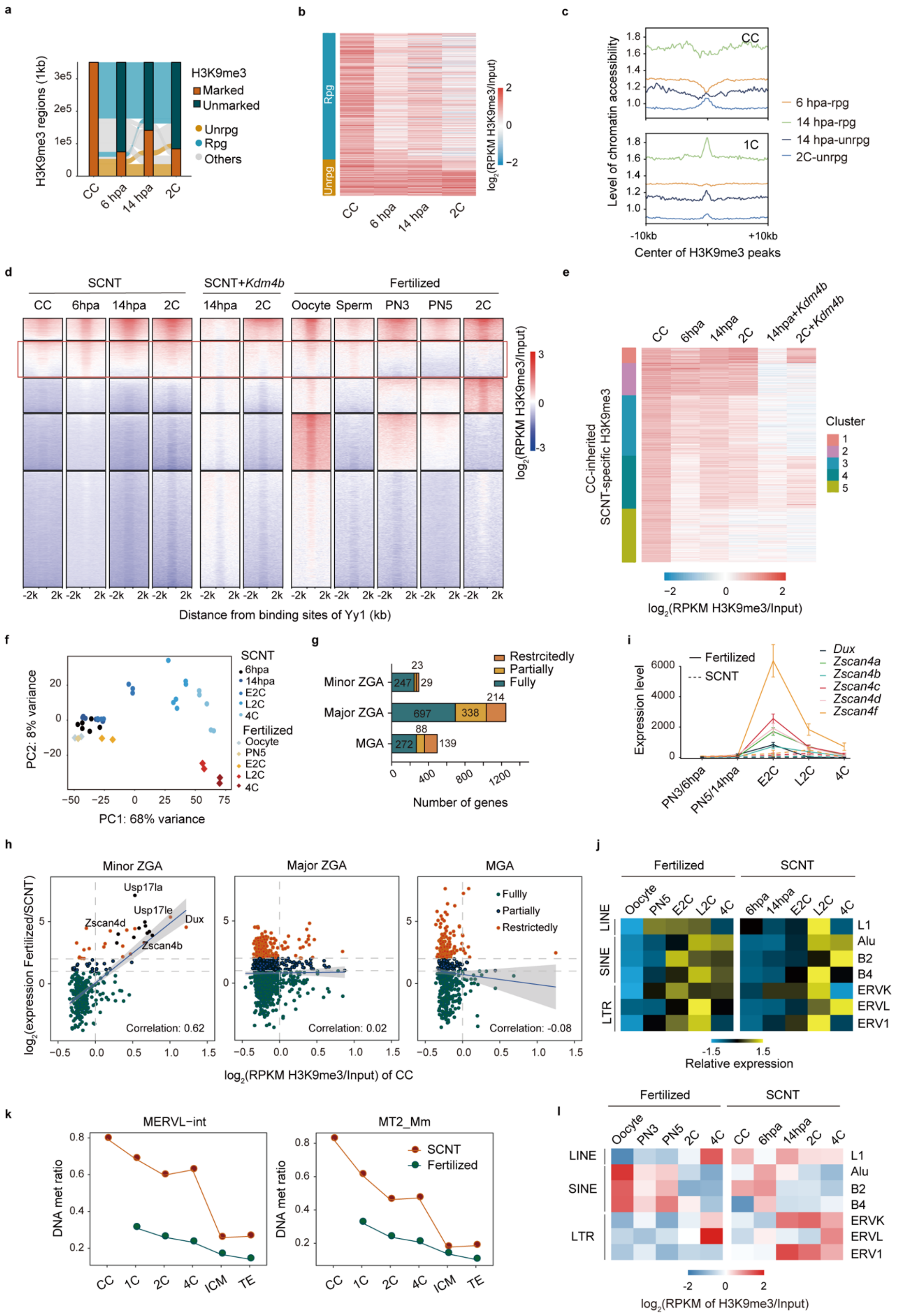

Supplementary Figure 3. Distinct chromatin states of rpgH3K9me3 and unrpgH3K9me3. (a) The dynamic of H3K9me3 peaks in SCNT embryos before 2C. UnrpgH3K9me3 regions are defined as regions with CC H3K9me3 retained after 14hpa. RpgH3K9me3 regions are defined as regions with CC H3K9me3 removed before 14hpa. **(b)** The H3K9me3 signal in SCNT embryos before 2C. The

rpgH3K9me3 and unrpgH3K9me3 regions were defined in **a**. **(c)** Chromatin accessibility profiler around the rpgH3K9me3 and unrpgH3K9me3 regions defined in **a**. Upper panel, DNase signal in CC; Lower panel, DNase signal in SCNT 1C embryo (data from GSE110851). **(d)** The H3K9me3 signal (log_2 transformed H3K9me3/Input ratio) around the YY1 binding regions. **(e)** The H3K9me3 signal in CC, SCNT and *Kdm4d* OE SCNT embryos. The regions were clustered by the H3K9me3 signal using the k-means function into 5 groups. **(f)** Principal component analysis (PCA) of the expression levels of replicates of fertilized and SCNT embryos. **(g)** The number of genes of minor ZGA, major ZGA, and MGA. The filling colors represent restrictedly (red), partially (orange), and fully (green) reprogrammed genes in SCNT, compared with the corresponding stage in fertilized embryos. **(h)** The association of H3K9me3 of CC and differential expression level (log2 transform foldchange) of minor ZGA (left), major ZGA (middle), and MGA (right) genes. Y-axis represents the log2 transformed ratio of the gene expression level of fertilized/SCNT at E2C, L2C, and 4C stages, respectively. X-axis represents the log2 transformed H3K9me3 signal in CC. The blue lines represent fitting Y ~ X to linear model, and the translucent ribbons represent a 95% confidence interval. The Pearson correlation coefficient is shown. **(i)** The expression level (normalized counts) of *Dux* and *Zscan4* family genes across SCNT and fertilized embryo developmental stages. The number of biologically independent samples: n=2 for the fertilized group (data from GSE71434); n=6 for the SCNT group. **(j)** The scaled mean expression level of LINE1, Alu, B2, B4, ERVK, ERVL, and ERV1. **(k)** DNA methylation ratio of MERVL-int and MT2_Mm across SCNT and fertilized embryo developmental stages (data from GSE108711). **(l)** The scaled mean H3K9me3 signal on LINE1, Alu, B2, B4, ERVK, ERVL, and ERV1 loci. **(i).** Data are presented as mean values ± SEM. Source data are provided as a Source Data file.

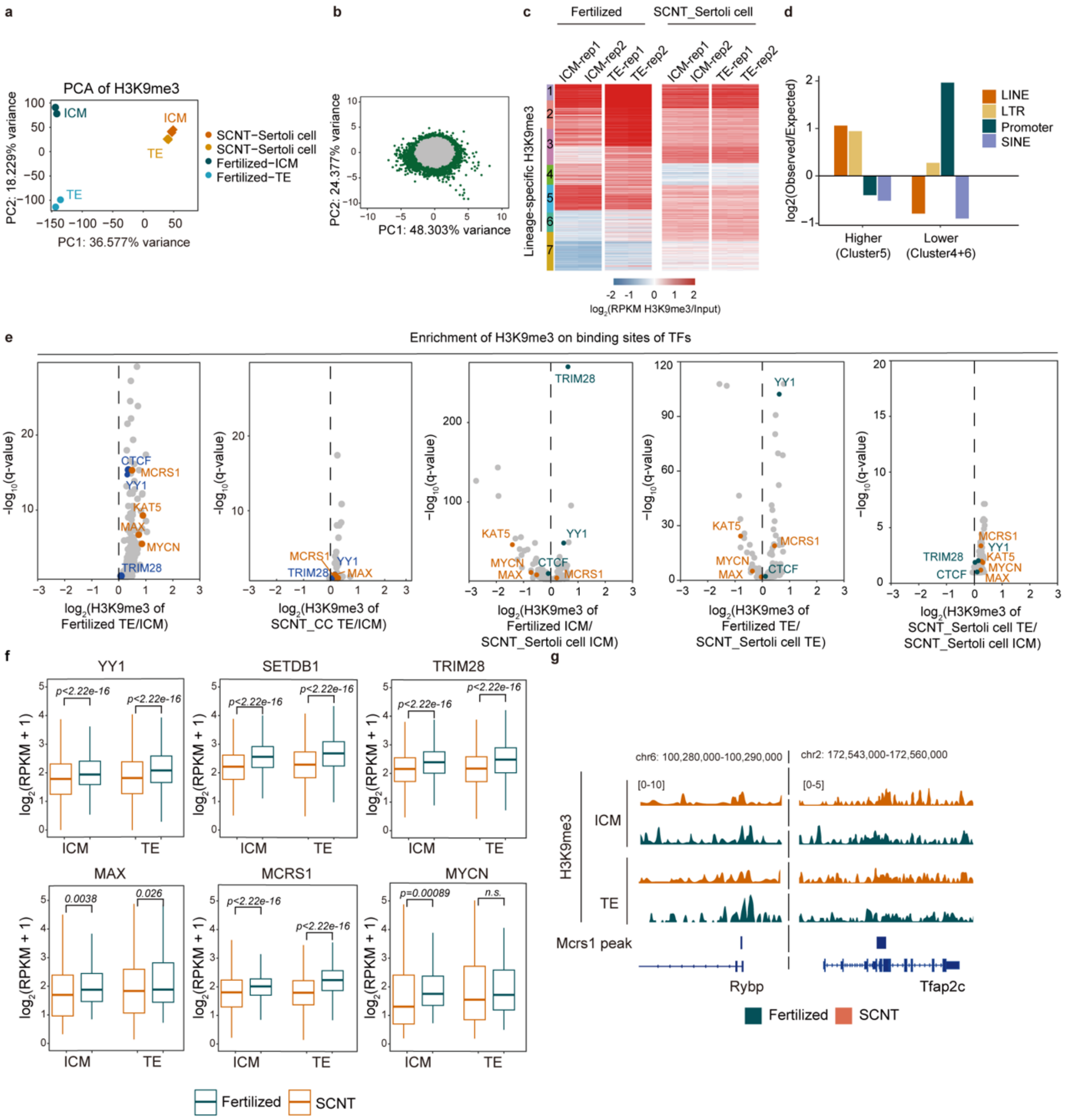

Supplementary Figure 4. Identification of potential regulatory factors that may be involved in the establishment of lineage-specific H3K9me3 in blastocysts. (a) PCA of H3K9me3 in ICM and TE of fertilized, cumulus cell-derived SCNT and Sertoli cell-derived SCNT embryos. **(b)** The eigenvectors on the first two principal components. Regions with the most positive and negative loading (green points, eigenvector value > 3, n = 3427) are screened out for cluster analysis (Figure **4b**). **(c)** The H3K9me3 signals in the ICM and TE of fertilized and Sertoli cell-derived SCNT embryos; the regions with the most positive and negative loading (n=3427, Supplementary Fig.**4a**) were classified into 7 clusters based on H3K9me3 signals using the k-means function. **(d)** The enrichment of H3K9me3 regions in LINE, LTR, SINE and promoter, these regions corresponding to the clusters in Figure **4b**. **(e)** The difference of H3K9me3 signal on TF binding sites between TE and ICM. The red plots represent the potential TFs that may be related to defective differential H3K9me3 deposition in SCNT blastocysts. The blue plots represent well-known chromatin architecture-related and H3K9me3-related factors. The gray plots represent other TFs. **(f)** H3K9me3 signals at binding sites of YY1, SETDB1, TRIM28, MAX, MCRS1 and MYCN in fertilized and cumulus cell-derived SCNT ICM/TE samples. n=2 biologically independent samples for the fertilized and SCNT groups (data from GSE97778). **(g)** H3K9me3 signals around the *Rybp* and *Tfap2c* loci in fertilized and cumulus cell-derived SCNT embryos. The signals are presented

as the RPKM values of H3K9me3. **(e).** Statistical significance was calculated with a two-tailed Fisher's exact test. **(f).** Statistical significance was calculated with a two-tailed Wilcoxon rank-sum test with BH multiple testing correction. ns, no significance. data are provided as a Source Data file.

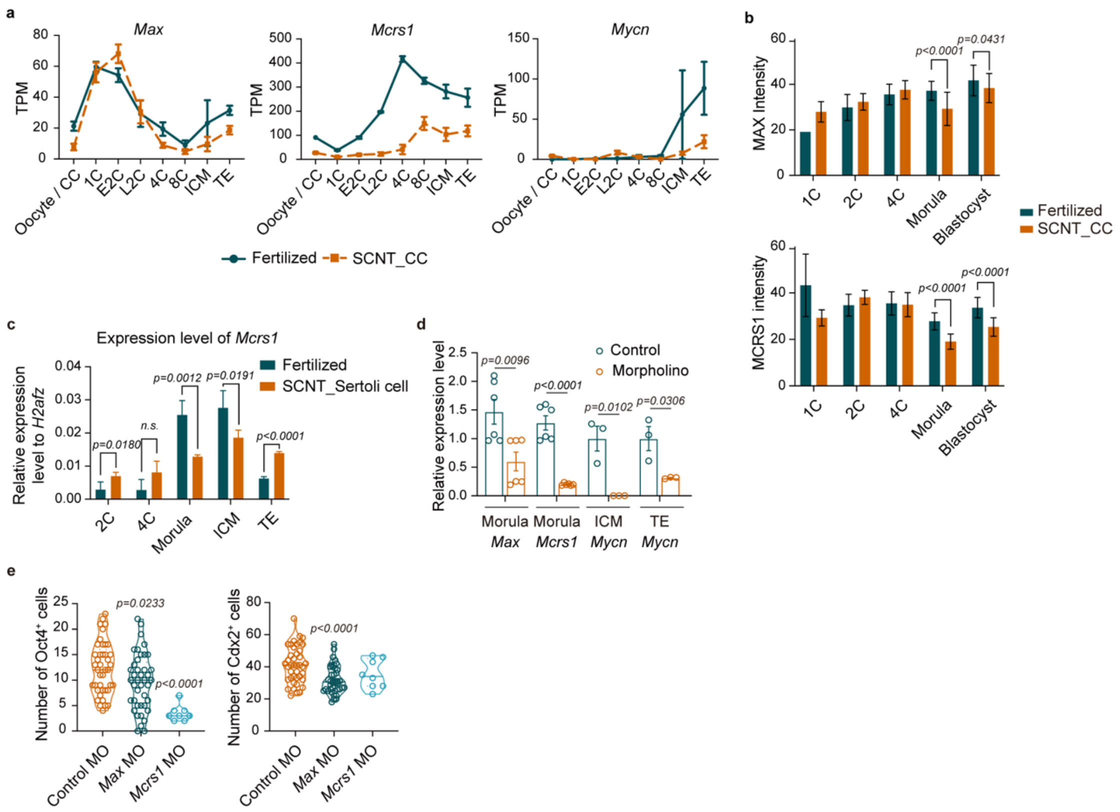

Supplementary Figure 5. Expression of Max and Mcrs1 were largely blocked by morpholino injection, severely harming blastocyst quality. (a) The variation trend of the expression level of *Max, Mcrs1,* and *Mycn* during pre-implantation development of fertilized (full line in green) and cumulus cell-derived SCNT (dotted line in red) embryos. Data are presented as the means ± SEM. The number of biologically independent samples: n=2 for oocyte, fertilized-1C, E2C, L2C, 4C and 8C from published data (GSE71434); n=4 for fertilized-ICM and TE; n=3 for CC and fertilized-morula; n=6 for SCNT-6hpa, E2C, L2C, 8C and morula; n=5 for SCNT-4C; n=9 for SCNT-ICM; n=10 for SCNT-TE. **(b)** Quantification of Max and Mcrs1 immunofluorescence signal in fertilized and cumulus cell-derived SCNT embryos. The number of counted nuclear at each stage in 2 biologically independent experiments: Max-Fertilized (n=1, 6, 8, 22, 38); Max-SCNT (n=3, 6, 7, 27, 29); Mcrs1-Fertilized (n=2, 6, 9, 26, 40); Mcrs1-SCNT (n=4, 6, 9, 21, 30). **(c)** The relative expression level of *Mcrs1* during Sertoli cell-derived SCNT embryo development. RT-qPCR analysis was performed in fertilized and Sertoli cell-derived SCNT embryos. The relative expression levels of *Mcrs1* relative to *H2afz* were compared between two groups at the same stage. **(d)** The relative expression level of the corresponding genes after morpholinos injection into fertilized embryos. RT-qPCR analysis was performed in morula for *Max* MO and *Mcrs1* MO groups, and in ICM and TE for the *Mycn* MO group. The relative expression levels of corresponding genes relative to *H2afz* were compared with control MO groups. Each point represents a single sample for RT-qPCR. The number of biologically independent samples: n=6 for Morula-*Max* and Morula-*Mcrs1*; n=3 for ICM-*Mycn* and TE-*Mycn*. **(e)** The number of $Oct4^+$ (left side) and $Cdx2^+$ (right side) cells in E3.5 blastocysts after injection of *Max* or *Mcrs1* morpholinos. Control MO, 41 blastocysts, 3 replicates; *Max* MO, 41 blastocysts, 3 replicates; *Mcrs1* MO, 8 blastocysts, 2 replicates. **(a, b, c).** Data are presented as mean values ± SEM. **(b, c, d, e).** Statistical significance was calculated with a two-tailed Student's t test. Source data are provided as a Source Data file.

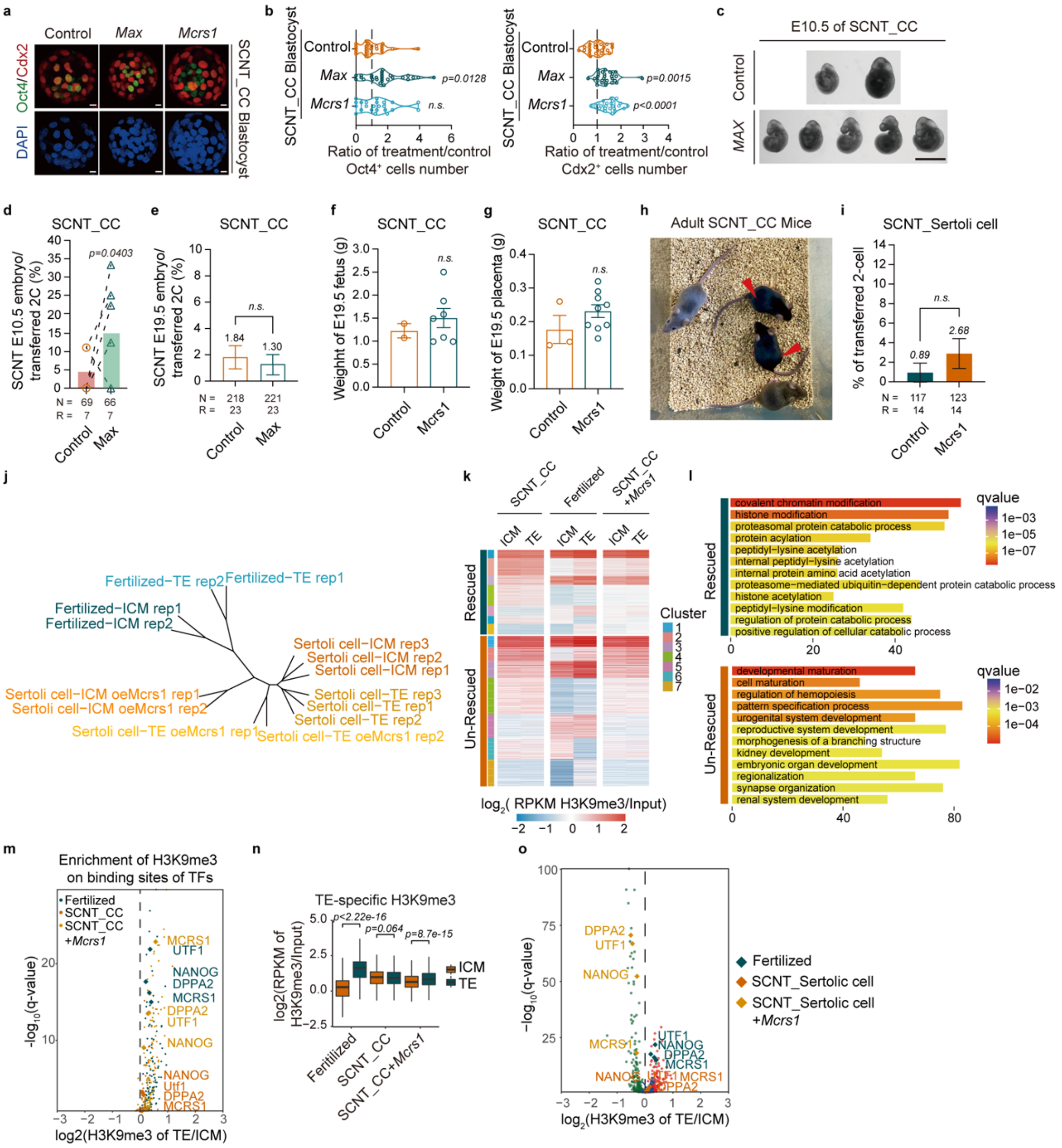

Supplementary Figure 6. *Max* and *Mcrs1* improve both pre- and post-implantation development of SCNT embryos.

(a) Immunostaining of Oct4 (green) and Cdx2 (red) in E4 cumulus cell-derived SCNT embryos after overexpression of *Max* or *Mcrs1*. DAPI stains for DNA (blue). Scale bars, 10μm. 3 experiments were repeated independently with similar results. **(b)** Violin plot showing an increased number of $Oct4^{+}$ (left side) and $Cdx2^{+}$ (right side) cells in E4 cumulus cell-derived SCNT blastocysts after overexpressing *Max* or *Mcrs1*. Control, 23 blastocysts, 3 replicates; *Max*, 28 blastocysts, 3 replicates; *Mcrs1*, 23 blastocysts, 3 replicates. **(c)** Representative images of E10.5 *Max* OE SCNT embryos. One representative result of at least three independent experiments is shown. Scale bars: 2 mm. **(d)** *Max* OE largely benefited the implantation ability of cumulus cell-derived SCNT embryos. N indicates the number of transferred 2-cell embryos. R represents the number of transferred pseudo-pregnant female mice. Spots connected by a dotted line stand for the same pseudo-pregnant female mice. **(e)** The birth rate of cumulus cell-derived SCNT full-term E19.5 fetuses after *Max* OE showed no significant difference from the control groups. N indicates the number of transferred 2-cell embryos. R represents the number of transferred pseudo-pregnant female mice. **(f, g)** The weight of cumulus cell-derived SCNT full-term E19.5 fetuses **(f)** and

placentae (**g**) after *Mcrs1* OE showed no significant difference from the control groups. 2 control fetuses and 6 *Mcrs1* OE fetuses are shown. 3 control placentae and 9 *Mcrs1* OE placentae are shown. **(h)** Representative healthy adult mice (pointed out by red arrows) generated by SCNT embryos with *Mcrs1* mRNA injection. **(i)** Bar plot showing the birth rate of full-term Sertoli cell-derived SCNT pups after *Mcrs1* OE. N indicates the number of transferred 2-cell embryos. R indicates the number of replicates. **(j)** Unrooted dendrogram of H3K9me3 signals in ICM and TE of fertilized and Sertoli cell-derived SCNT embryos (control and *Mcrs1* OE groups). **(k)** The H3K9me3 signals in the ICM and TE of fertilized, cumulus cell-derived SCNT and *Mcrs1* OE SCNT embryos. Rescued and Un-rescued are defined based on whether the H3K9me3 in *Mcrs1* OE SCNT blastocyst is closer to fertilized blastocyst than the control SCNT blastocyst, with the categories corresponding to the k-means clusters in Fig. **4b**. **(l)** Gene ontology analysis of the genes marked by H3K9me3 defined in **k**. **(m)** The enrichment of H3K9me3 on TF binding sites in ICM and TE of fertilized and cumulus cell-derived SCNT embryos (control and *Mcrs1* OE groups). **(n)** The relative H3K9me3 signal in fertilized and cumulus cell-derived SCNT blastocysts (control and *Mcrs1* OE groups) on the regions of TE-specific H3K9me3 (defined in fertilized TE). n=2 biologically independent samples for all groups. **(o)** The enrichment of H3K9me3 on TF binding sites in ICM and TE of fertilized and Sertoli cell-derived SCNT embryos (control and *Mcrs1* OE groups). **(e, f, g, i).** Data are presented as mean values ± SEM. **(b, d, e, f, g, i).** Statistical significance was calculated with a two-tailed Student's t test. **(n).** Statistical significance was calculated with a two-tailed Wilcoxon rank-sum test. **(m, o).** Statistical significance was calculated with a two-tailed Fisher's exact test. ns, no significance. Source data are provided as a Source Data file.

Supplementary Table 1. Sequences of siRNAs targeting *Suv39h2*.

Targeted gene	Sequence
Suv39h2-1	sense: 5’-ACCUUUGGAUGUUCAUGUATT -3’
	anti-sense: 5’- UACAUGAACAUCCAAAGGUGG -3’
Suv39h2-2	sense: 5’- GUAGAUAUUUGGUGGUUAATT -3’
	anti-sense: 5’- UUAACCACCAAAUAUCUACCG -3’
Suv39h2-3	sense: 5’- AGUUGUGUUGGCUUAUAAUTT -3’
	anti-sense: 5’- AUUAUAAGCCAACACAACUCC -3’

Supplementary Table 2. Sequences of primers to amplify cDNA of genes.

Genes	Sequence
Usp17la	forward:
	5'-TAATACGACTCACTATAGGATGGTGGTTGCTCTTTCCTT -3'
	reverse:
	5'-CTAGCAAACAAGCAGAAGCCTC -3'
Usp17lb	forward:
	5'-TAATACGACTCACTATAGGATGGTGGTTGCTC -3'
	reverse:
	5'-TCAGTTTGCAGTGCATATAGGTTGG -3'
Usp17lc	forward:
	5'-TAATACGACTCACTATAGGATGGTGGTTTCTCTTTCC -3'
	reverse:
	5'-CTAGCAAACAAGCAGAAGCCTCTGC -3'
Usp17ld	forward:
	5'-TAATACGACTCACTATAGGATGGTGGTTTCTCTTTCC -3'
	reverse:
	5'-CTAGCGAACAAGCAGAAGCTTCTGCCCTTG -3'
Usp17le	forward:
	5'-TAATACGACTCACTATAGGATGGTGGTTTCTC -3'
	reverse:
	5'-TCATCTCCCACCCTGACTACAGA -3'
Kdm4b	forward:
	5'- TAATACGACTCACTATAGGGAGAATGGGG
	TCCGAGGACCACAGCG -3'
	reverse:
	5'- CTAGAAGGGTGCTCCAGGCC -3'
Max	forward:
	5'-TAATACGACTCACTATAGGCCTGGGCCGTAGGAAATGAG-3'
	reverse:
	5'-GAAGGAGGATGCGACGAGAG -3'
Mcrs1	forward:
	5'- TAATACGACTCACTATAGGGCTTTGGGACTGTGAGAGGAT-3'
	reverse:
	5'- CCTCACTGTGGGGTGATCTT -3'

Supplementary Table 3. Targeting sites of Morpholinos.

Targeted gene	Morpholino targeting sites
Max	sense: 5’-CCGGGATGCCTGATGCAATATGAGA -3’
Mcrs1	sense: 5’-TGCCCAGCCCCTTACCTTCTTCTTC -3’
Mycn	sense: 5’-ACTCTACTCTAGGCTTTACCTGAGT -3’
Negative control	random 25N
