# Supplementary material for: Unreprogrammed H3K9me3 prevents minor zygotic genome activation and lineage commitment in SCNT embryos
Source: Nat Commun. 2023 Aug 9;14:4807. doi: 10.1038/s41467-023-40496-3 (PMC10412629; doi:10.1038/s41467-023-40496-3)
Supplement: Supplementary file 3 — Reporting Summary [file 41467_2023_40496_MOESM3_ESM.pdf]

nature portfolio

Corresponding author(s): Shaorong Gao

Last updated by author(s): Apr 24, 2023

Reporting Summary

Nature Portfolio wishes to improve the reproducibility of the work that we publish. This form provides structure for consistency and transparency in reporting. For further information on Nature Portfolio policies, see our Editorial Policies and the Editorial Policy Checklist.

Statistics

For all statistical analyses, confirm that the following items are present in the figure legend, table legend, main text, or Methods section.

n/a	Confirmed	
☐	☒	The exact sample size (*n*) for each experimental group/condition, given as a discrete number and unit of measurement
☐	☒	A statement on whether measurements were taken from distinct samples or whether the same sample was measured repeatedly
☐	☒	The statistical test(s) used AND whether they are one- or two-sided<br>*Only common tests should be described solely by name; describe more complex techniques in the Methods section.*
☐	☒	A description of all covariates tested
☐	☒	A description of any assumptions or corrections, such as tests of normality and adjustment for multiple comparisons
☐	☒	A full description of the statistical parameters including central tendency (e.g. means) or other basic estimates (e.g. regression coefficient) AND variation (e.g. standard deviation) or associated estimates of uncertainty (e.g. confidence intervals)
☐	☒	For null hypothesis testing, the test statistic (e.g. *F*, *t*, *r*) with confidence intervals, effect sizes, degrees of freedom and *P* value noted<br>*Give P values as exact values whenever suitable.*
☒	☐	For Bayesian analysis, information on the choice of priors and Markov chain Monte Carlo settings
☐	☒	For hierarchical and complex designs, identification of the appropriate level for tests and full reporting of outcomes
☐	☒	Estimates of effect sizes (e.g. Cohen's *d*, Pearson's *r*), indicating how they were calculated

Our web collection on statistics for biologists contains articles on many of the points above.

Software and code

Policy information about availability of computer code

Data collection	All sequence data were collected with the Illumina HiSeq X Ten or Nova platform.
Data analysis	Paired-end sequence data was trimmed adaptor and low-quality reads using cutadapt(v2.5). Then the filtered reads were processed using BWA (v0.7.17).The results (bigWig files) are shown in the track view generated with Integrative Genomics Viewer (IGV, v2.6.3). The H3K9me3 peaks were called with MACS2(v2.1.2) with the parameter "--broad" and over the input data.The enrichment of H3K9me3 peaks in annotated genomic regions was calculated using the Homer(v2.8) annotatePeaks command. The heatmap and profile line plot for the H3K9me3 signal around annotated genomic regions (i.e., CGIs, YY1 and H3K9me3 peaks) were generated using deepTools(v3.3.0).The genomic regions (i.e., LTRs, CGIs, promoters) with H3K9me3 modification were defined as those with the intersection of the H3K9me3 peak at the midpoint using the BEDtools(v2.30.0) intersect command with the parameters "-r -e -f 0.5". The TPM (transcripts per million) values of genes were obtained by StringTie(v2.0.3). Differential gene expression analysis was performed using DESeq2(v1.26.0). Functional enrichment analysis of gene sets was performed using the ClusterProfiler(v3.14.3).<br>Custom codes used for the analysis reported in this study are available at [https://github.com/rysterzhu/H3K9me3-in-SCNT].

For manuscripts utilizing custom algorithms or software that are central to the research but not yet described in published literature, software must be made available to editors and reviewers. We strongly encourage code deposition in a community repository (e.g. GitHub). See the Nature Portfolio guidelines for submitting code & software for further information.

Data

Policy information about availability of data

All manuscripts must include a data availability statement. This statement should provide the following information, where applicable:

- Accession codes, unique identifiers, or web links for publicly available datasets
- A description of any restrictions on data availability
- For clinical datasets or third party data, please ensure that the statement adheres to our policy

The H3K9me3 ULI-NChIP-seq data for oocytes, sperm, PN3, PN5, 2C, 4C, 8C, morula, ICM and TE of fertilized embryos were obtained from a previous publication26 (GSE97778), and the H3K9me3 ULI-NChIP-seq data for CC, 6hpa, 14hpa, 2C, 4C, 8C, morula, ICM and TE of SCNT embryos were generated in this study.
The RNA-seq data for oocytes, PN5, E2C, L2C, 4C, 8C and ICM of fertilized embryos were obtained from a previous publication88 (GSE71434). The RNA-seq data for SCNT embryos and other embryos were generated in this study.
The DNA methylation data for SCNT and fertilized embryos were obtained from a previous publication10 (GSE108711).
The ChIP-seq data of Mcrs1 in mESCs were obtained from a previous publication89 (GSE51746).
The raw sequence data reported in this paper have been deposited into the Gene Expression Omnibus (GEO). The accession number for the SCNT H3K9me3 ChIP-seq, CUT&RUN, and RNA-seq data generated in this paper is GSE195762.
Custom codes used for the analysis reported in this study are available at [https://github.com/rysterzhu/H3K9me3-in-SCNT].

Research involving human participants, their data, or biological material

Policy information about studies with human participants or human data. See also policy information about sex, gender (identity/presentation), and sexual orientation and race, ethnicity and racism.

Reporting on sex and gender	The study did not involve human participants
Reporting on race, ethnicity, or other socially relevant groupings	The study did not involve human participants
Population characteristics	The study did not involve human participants
Recruitment	The study did not involve human participants
Ethics oversight	The study did not involve human participants

Note that full information on the approval of the study protocol must also be provided in the manuscript.

Field-specific reporting

Please select the one below that is the best fit for your research. If you are not sure, read the appropriate sections before making your selection.

☒ Life sciences ☐ Behavioural & social sciences ☐ Ecological, evolutionary & environmental sciences

For a reference copy of the document with all sections, see nature.com/documents/nr-reporting-summary-flat.pdf

Life sciences study design

All studies must disclose on these points even when the disclosure is negative.

Sample size	Sample sizes were determined without statistical measures, but based on prior experience with the specific experiments and widely used sizes in relevant publications within this field of research in order to ensure that it will be appropriate for statistical analysis. See Methods for detail.
Data exclusions	No data were excluded from the analyses
Replication	All the sequencing experiments includes two or more independent biological replicates.
Randomization	No randomization was used in this study.
Blinding	Experiments execution, data collection and result analysis were usually carried out by the same person, therefore no blinding was used.

Reporting for specific materials, systems and methods

We require information from authors about some types of materials, experimental systems and methods used in many studies. Here, indicate whether each material, system or method listed is relevant to your study. If you are not sure if a list item applies to your research, read the appropriate section before selecting a response.

Materials & experimental systems

n/a	Involved in the study
☐	☒ Antibodies
☒	☐ Eukaryotic cell lines
☒	☐ Palaeontology and archaeology
☐	☒ Animals and other organisms
☒	☐ Clinical data
☒	☐ Dual use research of concern
☒	☐ Plants

Methods

n/a	Involved in the study
☐	☒ ChIP-seq
☒	☐ Flow cytometry
☒	☐ MRI-based neuroimaging

Antibodies

Antibodies used	primary antibodies against H3K9me3 (Active Motif, 39161), Max (Proteintech, 10426-1-AP), Mcrs1 (Sigma, HPA039057), Oct4 (Santa Cruz, sc-5279) or Cdx2 (Abcam, ab76541)
Validation	1. H3K9me3 (Active Motif, 39161)<br>https://www.thermofisher.cn/cn/zh/antibody/product/Histone-H3K9me3-Antibody-Polyclonal/39161<br><br>2. Max (Proteintech, 10426-1-AP)<br>https://www.ptglab.co.jp/Products/MAX-Antibody-10426-1-AP.htm<br><br>3. Mcrs1 (Sigma, HPA039057)<br>https://www.sigmaaldrich.cn/CN/zh/product/sigma/hpa039057<br><br>4. Oct4 (Santa Cruz, sc-5279)<br>https://www.scbt.com/p/oct-3-4-antibody-c-10/<br><br>5. Cdx2 (Abcam, ab76541)<br>https://www.abcam.cn/products/primary-antibodies/cdx2-antibody-epr2764y-ab76541.html

Animals and other research organisms

Policy information about studies involving animals; ARRIVE guidelines recommended for reporting animal research, and Sex and Gender in Research

Laboratory animals	The specific pathogen-free grade mice (SPF) grade mice, including C57BL/6j, DBA/2 and B6D2F1 mice were housed in the animal facility at Tongji University, Shanghai, China. The B6D2F1 hybrid mice (8-10 weeks old) were obtained from mating female C57BL/6j mice with male DBA/2 mice. All the mice had free access to food and water.
Wild animals	The study did not involve wild animals.
Reporting on sex	The study did not involve sex
Field-collected samples	The study did not involve samples collected from the field.
Ethics oversight	All experiments were performed in accordance with the University of Health Guide for the Care and Use of Laboratory Animals and were approved by the Biological Research Ethics Committee of Tongji University.

Note that full information on the approval of the study protocol must also be provided in the manuscript.

Plants

Seed stocks	The study did not involve plants
Novel plant genotypes	The study did not involve plants
Authentication	The study did not involve plants

ChIP-seq

Data deposition

☒ Confirm that both raw and final processed data have been deposited in a public database such as GEO.

☒ Confirm that you have deposited or provided access to graph files (e.g. BED files) for the called peaks.

Data access links
May remain private before publication.

https://www.ncbi.nlm.nih.gov/geo/query/acc.cgi?acc=GSE195762

Files in database submission

GSM5833522 6hpa_siSuv39h2_H3k9me3
GSM5833523 6hpa_control_H3K9me3
GSM5833802 cumulus cell-CC_H3K9me3_rep1
GSM5833803 cumulus cell-CC_H3K9me3_rep2
GSM5833804 cumulus cell-CC_H3K9me3_rep3
GSM5833805 cumulus cell-CC_H3K9me3_rep4
GSM5833806 NT-6h_H3K9me3_rep1
GSM5833807 NT-6h_H3K9me3_rep2
GSM5833808 NT-6h_H3K9me3_rep3
GSM5833809 NT-6h_H3K9me3_rep4
GSM5833810 NT-6h_H3K9me3_rep5
GSM5833811 NT-14h_H3K9me3_rep1
GSM5833812 NT-14h_H3K9me3_rep2
GSM5833813 NT-14h_H3K9me3_rep3
GSM5833814 NT-14h_H3K9me3_rep4
GSM5833815 NT-2cell_H3K9me3_rep1
GSM5833816 NT-2cell_H3K9me3_rep2
GSM5833817 NT-2cell_H3K9me3_rep3
GSM5833818 NT-2cell_H3K9me3_rep4
GSM5833819 NT-2cell_H3K9me3_rep5
GSM5833820 NT-2cell_H3K9me3_rep6
GSM5833821 NT-4cell_H3K9me3_rep1
GSM5833822 NT-4cell_H3K9me3_rep2
GSM5833823 NT-4cell_H3K9me3_rep3
GSM5833824 NT-8cell_H3K9me3_rep1
GSM5833825 NT-8cell_H3K9me3_rep2
GSM5833826 NT-8cell_H3K9me3_rep3
GSM5833827 NT-8cell_H3K9me3_rep4
GSM5833828 NT-8cell_H3K9me3_rep5
GSM5833829 NT-8cell_H3K9me3_rep6
GSM5833830 NT-8cell_H3K9me3_rep7
GSM5833831 NT-Morula_H3K9me3_rep1
GSM5833832 NT-Morula_H3K9me3_rep2
GSM5833833 NT-Morula_H3K9me3_rep3
GSM5833834 NT-Morula_H3K9me3_rep4
GSM5833835 NT-Morula_H3K9me3_rep5
GSM5833836 NT-Morula_H3K9me3_rep6
GSM5833837 NT-ICM_H3K9me3_rep1
GSM5833838 NT-ICM_H3K9me3_rep2
GSM5833839 NT-TE_H3K9me3_rep1
GSM5833840 NT-TE_H3K9me3_rep2
GSM5833841 Fertilized-PN5_H3K9me3_rep1
GSM5833842 Fertilized-PN5_H3K9me3_rep2
GSM5833843 cumulus cell-CC_input_rep1
GSM5833844 cumulus cell-CC_input_rep2
GSM5833845 cumulus cell-CC_input_rep3
GSM5833846 cumulus cell-CC_input_rep4
GSM5833847 NT-6h_input_rep1
GSM5833848 NT-6h_input_rep2
GSM5833849 NT-6h_input_rep3
GSM5833850 NT-6h_input_rep4
GSM5833851 NT-6h_input_rep5
GSM5833852 NT-14h_input_rep1
GSM5833853 NT-14h_input_rep2
GSM5833854 NT-14h_input_rep3
GSM5833855 NT-14h_input_rep4
GSM5833856 NT-2cell_input_rep1
GSM5833857 NT-2cell_input_rep2
GSM5833858 NT-4cell_input_rep1
GSM5833859 NT-4cell_input_rep2
GSM5833860 NT-4cell_input_rep3
GSM5833861 NT-8cell_input_rep1
GSM5833862 NT-8cell_input_rep2
GSM5833863 NT-8cell_input_rep3
GSM5833864 NT-8cell_input_rep4

GSM5833865 NT-Morula_input_rep1
GSM5833866 NT-Morula_input_rep2
GSM5833867 NT-Morula_input_rep3
GSM5833868 NT-Morula_input_rep4
GSM5833869 NT-Morula_input_rep5
GSM5833870 NT-ICM_input_rep1
GSM5833871 NT-ICM_input_rep2
GSM5833872 NT-TE_input_rep1
GSM5833873 NT-TE_input_rep2
GSM5833874 Fertilized-PN5_input_rep1
GSM5833875 Fertilized-PN5_input_rep2
GSM5849700 cumulus cell-CC_RNA_rep1
GSM5849701 cumulus cell-CC_RNA_rep2
GSM5849702 cumulus cell-CC_RNA_rep3
GSM5849703 NT-6h_RNA_rep1
GSM5849704 NT-6h_RNA_rep2
GSM5849705 NT-6h_RNA_rep3
GSM5849706 NT-6h_RNA_rep4
GSM5849707 NT-6h_RNA_rep5
GSM5849708 NT-6h_RNA_rep6
GSM5849709 NT-14h_RNA_rep1
GSM5849710 NT-14h_RNA_rep2
GSM5849711 NT-14h_RNA_rep3
GSM5849712 NT-14h_RNA_rep4
GSM5849713 NT-14h_RNA_rep5
GSM5849714 NT-14h_RNA_rep6
GSM5849715 NT-e2cell_RNA_rep1
GSM5849716 NT-e2cell_RNA_rep2
GSM5849717 NT-e2cell_RNA_rep3
GSM5849718 NT-e2cell_RNA_rep4
GSM5849719 NT-e2cell_RNA_rep5
GSM5849720 NT-e2cell_RNA_rep6
GSM5849721 NT-l2cell_RNA_rep1
GSM5849722 NT-l2cell_RNA_rep2
GSM5849723 NT-l2cell_RNA_rep3
GSM5849724 NT-l2cell_RNA_rep4
GSM5849725 NT-l2cell_RNA_rep5
GSM5849726 NT-l2cell_RNA_rep6
GSM5849727 NT-4cell_RNA_rep1
GSM5849728 NT-4cell_RNA_rep2
GSM5849729 NT-4cell_RNA_rep3
GSM5849730 NT-4cell_RNA_rep4
GSM5849731 NT-4cell_RNA_rep5
GSM5849732 NT-8cell_RNA_rep1
GSM5849733 NT-8cell_RNA_rep2
GSM5849734 NT-8cell_RNA_rep3
GSM5849735 NT-8cell_RNA_rep4
GSM5849736 NT-8cell_RNA_rep5
GSM5849737 NT-8cell_RNA_rep6
GSM5849738 NT-Morula_RNA_rep1
GSM5849739 NT-Morula_RNA_rep2
GSM5849740 NT-Morula_RNA_rep3
GSM5849741 NT-Morula_RNA_rep4
GSM5849742 NT-Morula_RNA_rep5
GSM5849743 NT-Morula_RNA_rep6
GSM5849744 NT-ICM_RNA_rep1
GSM5849745 NT-ICM_RNA_rep2
GSM5849746 NT-ICM_RNA_rep3
GSM5849747 NT-ICM_RNA_rep4
GSM5849748 NT-ICM_RNA_rep5
GSM5849749 NT-ICM_RNA_rep6
GSM5849750 NT-TE_RNA_rep1
GSM5849751 NT-TE_RNA_rep2
GSM5849752 NT-TE_RNA_rep3
GSM5849753 NT-TE_RNA_rep4
GSM5849754 NT-TE_RNA_rep5
GSM5849755 NT-TE_RNA_rep6
GSM5849756 NF-ICM_control_RNA_rep1

	GSM5849757 NF-ICM_control_RNA_rep2<br>GSM5849758 NF-ICM_mphMax_RNA_rep1<br>GSM5849759 NF-ICM_mphMax_RNA_rep2<br>GSM5849760 NF-ICM_mphMcrs1_RNA_rep1<br>GSM5849761 NF-ICM_mphMcrs1_RNA_rep2<br>GSM5849762 NF-TE_control_RNA_rep1<br>GSM5849763 NF-TE_control_RNA_rep2<br>GSM5849764 NF-TE_mphMax_RNA_rep1<br>GSM5849765 NF-TE_mphMax_RNA_rep2<br>GSM5849766 NF-TE_mphMcrs1_RNA_rep1<br>GSM5849767 NF-TE_mphMcrs1_RNA_rep2
Genome browser session (e.g. UCSC)	NA

Methodology

Replicates	Two or more replicates were adopted.
Sequencing depth	About 80 million reads pairs for each replicate.
Antibodies	As described above.
Peak calling parameters	macs2 callpeak --broad -t $ddir/${k}_K9me3.sorted.bam -c $ddir/${k}_input.sorted.bam --outdir $odir -f BAMPE -g mm -n $k
Data quality	See Supplementary Figures.
Software	BWA mem; MACS2
